# Talking mental health - study protocol for a cluster-randomized controlled trial of mental health prevention in elementary schools in Germany

**DOI:** 10.3389/fpsyg.2025.1658196

**Published:** 2026-01-12

**Authors:** Sophia Peter, Julia Bednarz, Paula Sommer, Svenja Taubner

**Affiliations:** 1 Institute for Psychosocial Prevention, Heidelberg University Hospital, Heidelberg, Germany; 2 Institute of Psychology, Heidelberg University, Heidelberg, Germany; 3 Medical Faculty, Heidelberg University, Heidelberg, Germany; 4 Alexianer Hospital Cologne, Cologne, Germany

**Keywords:** elementary school, help-seeking, mental health, mental health literacy, prevention, stigma

## Abstract

**Background:**

Mental disorders are highly prevalent, and mental health problems often start in childhood or adolescence. However, barriers to care, such as low mental health literacy and stigma, increase the risk of developing mental disorders. The abilities to recognize, talk about and seek help when mental health problems occur are important processes to overcome such barriers. The school-based prevention program “Talking mental health” (TMH) aims to reduce stigma among children in elementary schools and their families by increasing mental health literacy and help-seeking behavior.

**Methods:**

In this study, the “Talking mental health” (TMH) prevention program is evaluated using a cluster-randomized design, comparing an intervention group with a waitlist control group. The primary outcomes are mental health literacy, stigma and help-seeking behavior. Measurement takes place pre and post, as well as at a six-week follow-up. The sample size aims for 35 school classes with approximately 525 parents and children. The study also examines the effects of an additional parent training on children’s and parents’ mental health.

**Discussion:**

If effective, this study provides a validated prevention program to promote mental health in elementary schools, potentially allowing wider implementation. This could help families access existing support structures at an early stage, therefore preventing the chronic progression of mental health problems. The methodological and practical challenges of the study are discussed, as well as general challenges of school-based prevention efforts.

**Clinical trial registration:**

https://drks.de/search/en/trial/DRKS00035171, identifier DRKS00035171.

## 1 Introduction

Both parents and children express uncertainty about how to recognize mental health problems and where to find support (Reardon et al., 2017; Tharaldsen et al., 2017). Mental health literacy is an important health competence and is defined as knowledge and beliefs about mental disorders that facilitate their recognition, management or prevention (Jorm et al., 1997). The importance of mental health literacy is highlighted by the high prevalence of mental disorders in the general population. In Germany, where this study takes place, 42.6% of people suffer from a mental disorder at least once in their life (lifetime prevalence in Germany; Jacobi et al., 2004). Children and adolescents are also affected. Half of mental disorders have their onset around the age of 14 (Kessler et al., 2005). The median age of manifestation for anxiety and impulse-control disorders is even earlier, at 11 years (Kessler et al., 2005). With respect to prevalence rates in the UK, 20% of all children struggle with mental health problems by the time they finished elementary school (Morrison Gutman et al., 2015). Across Europe, the prevalence of mental disorders among 5-18-year-olds was found to be 15.5% (Sacco et al., 2024). In Germany, prevalence rates of 17.6% were reported (Barkmann and Schulte-Markwort, 2012). Additionally, the coronavirus pandemic has seemingly exacerbated the situation, especially for children (Ravens-Sieberer et al., 2022).

Furthermore, mental disorders during adolescence are associated with numerous negative long-term outcomes, such as unfavorable educational burdens, financial difficulties, poor social functioning and increased risk of problematic substance use (e.g., Shaw et al., 2012; Asselmann et al., 2018). Even though mental disorders are highly prevalent and associated with negative consequences, only a minority of those affected receive professional treatment (Hintzpeter et al., 2015). The waiting times for outpatient psychotherapy are long (in Germany, approximately 20 weeks from initial contact to the start of treatment), and children and adolescents face even longer waiting times than older patients do (Singer et al., 2022). Moreover, children and adolescents are largely dependent on their parents in seeking help. Preventive intervention in settings that reach as many children as possible, such as schools, has an important role in changing low early access to care. Students themselves expressed a desire for more proactive education and understanding of mental health in schools (Marinucci et al., 2022). Given the early onset of mental disorders, this study protocol presents an approach to mental disorder prevention with the aim of increasing mental health literacy and help-seeking behavior in elementary schools using age-appropriate materials. The following sections highlight the barriers that young people face in seeking help and emphasize the crucial role of mental health literacy and stigma in this process.

A systematic review examined the most frequently mentioned barriers preventing young people from seeking help (Radez et al., 2021). The two most cited categories were individual and social factors (Radez et al., 2021). Individual factors included young people not knowing whether their problem was severe enough (e.g., Guterman et al., 2010) and not knowing where to find help (e.g., Sheffield et al., 2004). Overall, young people were found to have little knowledge of available help services for mental disorders (Tharaldsen et al., 2017). Indeed, seeking help was often seen as a weakness (e.g., Tharaldsen et al., 2017), and young people believed that they could handle it on their own (e.g., Sheffield et al., 2004; Rickwood et al., 2005) or hoped that it would go away if they did not talk about it (e.g., Guterman et al., 2010). Among social factors, the most commonly reported barrier was perceived stigma (e.g., Gronholm et al., 2017), accompanied by fear of losing one’s status within a peer group (e.g., Tharaldsen et al., 2017) or being insulted and ostracized (Gronholm et al., 2017). Experiencing stigma for mental health problems is not only a fear but also a reality for children and adolescents (Kaushik et al., 2016). It is therefore not surprising that perceived public stigma was associated with lower intentions to seek help among adolescents (Nearchou et al., 2018).

The process model by Rickwood and colleagues suggests that four steps are required to seek help for mental health problems: (1) awareness and appraisal of the problem (emotional competence); (2) telling others that there is a problem (expressing symptoms and need for support); (3) the source of help must be available; and (4) willingness to open up to the source of help (Rickwood et al., 2005). Emotional competence in step one is defined as the ability to recognize, describe, understand and functionally manage emotions and has been associated with increased help-seeking behavior (Rickwood et al., 2005). Each of these four steps can be affected by perceived barriers (Rickwood et al., 2005). Therefore, reducing such barriers, promoting mental health literacy and emotional competence are key to increasing help-seeking behavior. For example, studies have shown that fewer perceived barriers (such as stigma) are associated with more formal and informal help-seeking behavior (Sheffield et al., 2004; Zuaboni et al., 2021). Mental health literacy was also found to be associated with increased help-seeking behavior (e.g., Gorczynski et al., 2017). Additionally, findings highlight an inverse relationship between mental health literacy and stigma: greater knowledge of mental disorders was associated with more positive attitudes toward help-seeking behavior (Sheffield et al., 2004).

While the literature agrees that mental health literacy and stigma play crucial roles in the process of considering help-seeking behavior, there is disagreement regarding whether these are independent factors or whether stigma acts as a mediating factor. Several models suggest that mental health literacy, or knowledge about mental health disorders, leads to more positive (i.e., less stigmatizing) attitudes, which in turn facilitate help-seeking behavior (Sheffield et al., 2004; Jung et al., 2017; Ma et al., 2023b). However, some studies found a direct effect of mental health literacy on help-seeking behavior without mediation through stigma (Sheffield et al., 2004; Jung et al., 2017), whereas others demonstrated that reducing stigma through increased mental health literacy can increase help-seeking behavior (Kim, 2023; Yang et al., 2024).

In summary, interventions that aim to improve mental health should focus on reducing stigma among young people and their families while at the same time increasing mental health literacy (Xu et al., 2018). Children and adolescents should be equipped with the knowledge and confidence to identify mental health problems, talk about them, access appropriate support, and help their peers effectively. Parents should also be informed about when and where to seek help for their children’s mental health problems and how to discuss these issues openly. Social support has been shown to be an important factor in young people’s help-seeking behavior, independent of mental health literacy and stigma reduction (Rickwood et al., 2005; Jung et al., 2017; Yang et al., 2024). Therefore, interventions should not only target children but also include their parents.

A recent review of randomized controlled trials testing school-based interventions to address mental health literacy and mental health stigma found an overall moderate level of evidence (Ma et al., 2023a). However, they identified only two randomized controlled trials in elementary schools (Pitre et al., 2007; Link et al., 2020), one of which included children in grade 6 (age 11.5; Link et al., 2020). This highlights the urgent need for evidence-based interventions for children under 10 years of age. Furthermore, a review by the same authors revealed no strong evidence for the effectiveness of mental health literacy interventions on help-seeking behavior (Ma et al., 2023b). On the basis of this review, the authors expressed the need for practical and targeted help-seeking content in school-based mental health literacy interventions if the aim is to increase help-seeking behavior (Ma et al., 2023b). In conclusion, this meta-analysis highlighted the positive effects of mental health literacy interventions in elementary schools on increased mental health literacy. However, only studies with children aged 10 years and over were included in the analysis. In contrast, the effects of mental health literacy interventions on stigma reduction and help-seeking behavior are less clear across studies (Ma et al., 2023b). The available studies show that there is an urgent need for evidence-based interventions in elementary schools that address not only mental health literacy but also help-seeking behavior and stigma.

The Anna Freud National Center’s prevention program “Talking mental health” (TMH) is one of the few such intervention programs addressing both mental health literacy and help-seeking behavior, which is also aimed at elementary school children aged 8–10 years (Sharples et al., 2017). Although the program has been widely implemented in the UK, it has not been extensively evaluated. An initial non-control group survey revealed that the training was very well received and that the children wanted to talk more about their feelings after the training (Sharples et al., 2017). The culturally adapted and tailored version for German-speaking countries, carried out by college students, will be fully evaluated as part of this research project.

Despite the positive impacts mentioned above, increasing mental health literacy does not directly reduce existing symptoms or disability (e.g., Jorm et al., 1997). Therefore, prevention programs require complimentary and targeted interventions to alleviate psychological symptoms in children and parents under stress. Parent training programs offer a cost-effective solution, as parental factors have been found to play a crucial role in the development of mental health problems in children (e.g., Yap et al., 2014). Evidence suggests that parenting skills training (Emotion focused skills training for parents; EFST-P; Dolhanty et al., 2022) leads to a reduction in both internalizing and externalizing symptoms in children (Ansar et al., 2022; Zahl-Olsen et al., 2023). In addition, immediately after the training and at follow-ups 4, 8 and 12 months later, parental self-efficacy and emotion regulation were strengthened, and psychological distress decreased in both parents and children (Ansar et al., 2024). Emotion focused skills training for parents (EFST-P) focuses on developing the following key skills: (1) validating children’s feelings, (2) increasing motivation, (3) apologizing, when necessary, and (4) setting appropriate boundaries for children (Dolhanty et al., 2022). In this study, all children receive the universal prevention program “Talking mental health” (TMH), and families with stress are also offered Emotion focused skills training for parents (EFST-P).

This research project investigates whether the school-based prevention program “Talking mental health” (TMH) can increase mental health literacy and help-seeking behavior while reducing the stigma of mental disorders. The primary aim of the prevention project is to improve children’s knowledge and attitudes toward managing difficult emotions. Parents are supported to talk to their children about their feelings and, if necessary, to access appropriate support services. In addition, the effects on college students implementing and being trained in the program are examined.

We expect that children who have completed the TMH program (compared to those in the waitlist control group) will show improved knowledge and skills in managing mental health problems and difficult emotions (increased mental health literacy; Hypothesis 1a), greater willingness to talk about their difficult feelings (increased intentional help-seeking behavior, Hypothesis 1b), increased actual help-seeking behavior (Hypothesis 1c), and reduced stigmatization of mental health problems (Hypothesis 1d). We expect these effects to be present both 1 week after the intervention and at least 6 weeks later. We expect that in the intervention group, an increase in mental health literacy will lead to a decrease in stigma, which in turn will cause an increase in help-seeking behavior (Hypothesis 1e).

Additionally, we expect that parents of children who completed the prevention program (compared with parents in the waitlist control group) will show increased help-seeking behavior for their children (Hypothesis 2a) and reduced stigmatization of mental health problems (Hypothesis 2b).

College students trained in and implementing the TMH program should show less stigmatization toward others than should college students in the control group (Hypothesis 3).

Finally, we expect parents who participate in the parent training program (Emotion focused skills training; EFST-P) and their children to demonstrate improved mental health after the conclusion of the program (Hypothesis 4a). This effect is achieved by increasing parents’ self-efficacy (Hypothesis 4b) and mentalization skills (Hypothesis 4c).

## 2 Methods and analysis

### 2.1 Design and randomization

This study evaluates the “Talking mental health” prevention program using cluster randomization with a pre-post follow-up measurement approach. Elementary schools are randomly assigned to either the experimental condition or the waitlist control group by the study coordinator (SP) using digital coin toss^1^. The assignment is documented in an electronic file. Neither stratification nor block randomization are applied. The participants in the experimental condition are instructed to complete a questionnaire just before the prevention program. The post-measurement takes place at least 1 week after the intervention, and the follow-up at least 6 weeks later. In comparison, all three measurements in the waitlist control group are taken at the same time intervals, but the intervention takes place only after the last measurement.

A quasi-experimental design is used for the group of college students who carry out the prevention program. We compare students from the student organization “Vitaphilie” (Medical Students’ Association of the Heidelberg University), who are trained to deliver the program, with students from other medical student organizations not trained in the program. The students assign themselves to the student organizations independently (quasi-experimental; students carrying out the training vs. students from other student organizations).

Upon request and if needed, a psychological counseling session for the parents and subsequent parent training are offered after completion of the project (parent skills training based on EFST-P; Dolhanty et al., 2022). Parents are surveyed before and after the training, and 4 months later, and compared with parents who did not receive the training (quasi-experimental allocation).

### 2.2 Participants and recruitment

Elementary schools in and around Heidelberg (Germany) are invited to participate in the intervention study. Staff from our institute attended the annual conference of headmasters from elementary schools in Heidelberg and the Rhine-Neckar region. In addition, the 28 elementary schools in Heidelberg received written invitations and information about the intervention. A sample size of at least 35 classes, with 15 participating children in each class, is planned. The sample consists of elementary school children in grades 3 and 4 (approximately 8–10 years old) and their parents. Approximately 35 teachers, 45 college students implementing the prevention program and a similar number of control college students are also assessed. The inclusion criterion for the study is sufficient knowledge of written and spoken German and informed consent, with parents providing consent for their children. Teachers and parents are informed about the study both in writing (information letter) and orally (at a parents’ school evening). Children with special educational needs are included in our sample if they have sufficient German language skills to read and understand the questionnaires. If needed, children will be supported in reading the questionnaires by an integration assistant, a teacher, or a member of the project staff.

A second recruitment phase takes place after the third survey. Parents who have indicated that they may be contacted again are invited to the counseling session, parent training and follow-up surveys. For this part of the study, parents provide informed consent online.

The students carrying out the TMH program are part of a student organization of the Medical Student Council of the Heidelberg University. “Vitaphilie” is a group whose aim is to raise awareness and destigmatize mental illness. The student organization consists mainly of medical students and psychology students. The project is coordinated by the Institute for Psychosocial Prevention, which is part of the Center for Psychosocial Medicine at Heidelberg University Hospital.

### 2.3 Power

The sample size of the study was determined by taking into account recommendations based on model analyses, which suggested that 35 classes of 15 students were needed to measure a moderate effect in a three-level design (Kerkhoff and Nussbeck, 2019).

### 2.4 Study procedures

An overview of the respective assessments and intervention dates can be found in Table 1. Children and students are assessed three times, parents three to six times, and teachers once. The surveys for parents, children, teachers, and students each take approximately 20–30 min. The survey for the children takes place onsite at their school and is supervised by trained staff members. The children complete the questionnaires using tablets provided by the school (online version). If tablets are not available at the school, paper-and-pencil questionnaires are used instead. Surveys for parents, students, and teachers are available online, with the participants receiving a link to the online questionnaires via email.

**Table 1:** Schedule of enrollment, interventions, and assessments.

Timepoint	Enrollment	Post-allocation					
	−t_1_	t_1_	t_2_	t_3_	t_4_	t_5_	t_6_
Eligibility screen, informed consent and allocation	X				X		
Interventions							
Talking mental health (IG)		X					
Talking mental health (CG)				X			
Emotion focused skills training					—		
Assessments							
*Parents*: stigma (PMHSS), help-seeking (GHSQ & AHSQ), mental health parents (PHQ-4), mental health children (KIDSCREEN-10), mentalizing (PRFQ)^1^, parental self-efficacy (FSW)^1^		X	X	X	X	X	X
*Children*: stigma (PMHSS), mental health literacy (vignettes), mental health (KIDSCREEN-10), help-seeking (GHSQ & AHSQ), satisfaction		X	X	X			
*College Students*: stigma (PMHSS & OMS-HC), satisfaction		X	X	X			
*Teachers*: stigma (PMHSS), satisfaction				X			

IG, Intervention group; CG, Control group; PMHSS, Peer Mental Health Stigmatization Scale; PHQ-4, Patient Health Questionnaire; KIDSCREEN-10; GHSQ, General Help-Seeking Questionnaire; AHSQ, Actual Help-Seeking Questionnaire; OMS-HC, Opening Minds Stigma Scale for Health Care Providers; PRFQ, Parental Reflective Functioning Questionnaire; FSW, Parenting self-efficacy questionnaire.

1 Only at t_4_, t_5_, and t_6_.

To implement the project in elementary schools in Heidelberg, approval was obtained from the relevant school authorities. In addition, school administrations must agree that the prevention program and surveys can be carried out. Project staff also attend parent-teacher conferences and student body meetings to provide parents, teachers and college students with detailed information about the program. The project staff presents the project and the data collection process. Approximately 1 week later, signed consent forms are collected from the schools and students. After the parents’ school evening, all parents receive the link to the first survey (t_1_). The children in the intervention group are assessed for the first time immediately prior to the intervention (t_1_), then at least 1 week later (t_2_) and again 6 weeks later (t_3_). For children in the control group, the first assessment is scheduled after the parent-teacher conference (t_1_), 1 week later (t_2_), and at least 6 weeks later (t_3_). In the control group, the final assessment takes place just before the TMH prevention program. At the same time as the child assessments, parent assessments 2 and 3 are sent by email. Following the prevention program, teachers also receive an invitation to participate in their survey.

After the third survey, parents can request feedback based on their assessment of whether their child’s mental health is below average, average or above average compared with other children of the same age. Parents can also provide their email address if they like a psychological consultation. The consultation is conducted by a psychologist (SP) and lasts 50 min. During the consultation, the current problem situation is recorded, information about appropriate help is given, and the parent training is introduced and offered. The parent training takes place afterwards. Parents are assessed again before the parent training (t_4_), after the parent training (t_5_), and again 4 months later (t_6_). To take part in t_4_ to t_6_, parents must give their informed consent online. For this part of the study, parents who indicated in the first consent form that they would like to be contacted again are contacted. Parents who take part in the parent training and those who do not, can take part in these three additional surveys.

Finally, college students who participate in Vitaphilie are recruited at the beginning of each semester. At this point in time, both the intervention group and the control group receive the first survey. In the middle of the semester, the control group completes the second questionnaire, whereas each person in the Vitaphilie group receives an email with the post-intervention survey after completing the prevention program. Both groups of students complete the follow-up questionnaire at the end of the semester.

### 2.5 Intervention

#### 2.5.1 Talking mental health

“Talking mental health” is a school-based prevention program addressing 3rd and 4th graders. The aim of the intervention is to raise awareness of mental health by covering different types of feelings, teaching children how to talk about and manage their feelings, when to seek help and how to support others.

TMH uses child-friendly material to cover the following topics: (a) what is mental health, (b) what are “small” (everyday feelings) and “big” (feelings that last long and impact daily life and mental health) feelings, (c) how can I deal with these feelings, (d) how can I talk about difficult feelings, (e) where can I get help, and (f) how can I be a good listener when someone approaches me? The program is delivered in a one-day workshop during school hours in the classroom (4 school hours) by 4 to 5 medical or psychology students (facilitators). The class teacher is present to support the facilitators.

The content is introduced to the children through a short video (5 min, English version^2^ and German version^3^). The video tells the story of 10-year-old Luca, who faces emotional challenges, by describing their feelings and offering possible coping strategies, as well as showing how seeking help can contribute to mental health. During the session, small parts of the video are shown and discussed with the children. To create a safe and private environment, each facilitator works with 5–6 children at an individual table. Each table group has a “mood barometer,” which is used to check the children’s emotional state. Each child is also given a handout called “My Mental Health License” to take home. Throughout the session, each aspect is marked down in there. The program also includes a section where possible points of contact, both informal and formal, are worked out with the children. In addition to the video, other materials are used to work on the themes mentioned above. For example, the distinction between “small” and “big” feelings can be explored by sorting statements and brainstorming personal experiences. The children also create their own support circle. They practice raising difficult topics by taking turns using conversation starters with their peers. Role play is also used to practice validating the feelings of others through attentive listening. Parents are provided with online information material to guide them on the following topics:

(1) How do I talk to my child about difficult feelings and mental health?
(2) Where can I go for professional support for my child in and around Heidelberg?

The material on “How do I talk to my child about difficult feelings and mental health?” were developed using recommendations for communicating with children (Döpfner, 2019) and existing material from the Anna Freud National Center for Children and Families (2025), e.g., how to start a conversation with children about feelings and general recommendations (curiosity, staying calm, open questions, taking children seriously, praise). The material on “Where can I go for professional support for my child in and around Heidelberg?” were developed by looking at existing help centers in Heidelberg, Germany. Parents were given information on which (professional) contact point is suitable for which difficulties, e.g., the difference between a psychiatrist and a psychotherapist.

TMH was originally developed by the Anna Freud National Center for Children and Families (Sharples et al., 2017). In 2019, our research team translated and adapted the intervention for Germany through a pilot study. In contrast to the original program, our program is not carried out by the teacher but by college students. A systematic review found that teachers frequently report stress when simultaneously acting as educator and program facilitator (Schäfer et al., 2025). In addition, a lack of time represents a common barrier keeping teachers from implementing prevention programs (Schäfer et al., 2025). Fortunately, students from the “Vitaphilie” working group were available and interested in cooperating with us. This allowed us to assign several facilitators to a class, with each student serving as the contact person for a group of five to seven children. Previous research has examined the involvement of peer facilitators in health education and has shown that this approach can help fill gaps on an equal footing that professionals are sometimes unable to reach (Dodd et al., 2022). This approach was also favored by the schools we recruited so far. Our adaptation lasts 4 school hours (3–3.5 h), whereas the original program takes 1–1.5 h. It has been suggested that interactive methods facilitate learning in mental health prevention programs (Schäfer et al., 2025). Therefore, we decided to explore the different topics in greater depth, with the use of more didactic methods and materials (e.g., the mood barometer, the role play). To include this, we had to extend the program while also making sure it will not be perceived as too lengthy (Schäfer et al., 2025). In line with the recommendations of Ma et al. (2023b), our program provides more detail about possible support services, such as doctors, psychologists, and therapists, and promotes the German helpline for children (“Nummer gegen Kummer”). The original prevention program did not include practical, targeted help-seeking content. Another extension is the introduction of a “toolbox” in which children can collect skills for dealing with small feelings. Other research projects have also recommended the inclusion of specific strategies for dealing with emotions (Kuyken et al., 2022). As additional interactive content, we have included an activity in which the whole class physically acts out different feelings, e.g., disgust, anger, and happiness.

#### 2.5.2 Emotion focused skills training

We offer parent training based on the Emotion focused skills training for parents (EFST-P). The EFST-P is a manualized group program for parents (Dolhanty et al., 2022). It uses an emotion-processing, skill-based approach that provides parents with simple, practical tools that they can use immediately in their families. The EFST-P can be delivered in different ways (full-day workshop or weekly group meetings; Dolhanty et al., 2022). In the present study, 6 weekly group sessions of 100 min each are conducted by two psychologists. The aim of the program is to overcome feelings of shame, guilt or fear that interfere with effective parenting. Parents learn to navigate their emotions by understanding children’s emotions and needs. The EFST-P focuses on developing the following four key skills:

(1) Validating children’s feelings: Instead of trying to solve the child’s problems quickly, parents are taught to recognize the child’s feelings and put them into words. This should help the child process their feelings and calm down.
(2) Increasing motivation: Parents learn why they find it difficult to understand and validate their child’s feelings. So-called emotional traps are introduced, which can prevent parents from being emotionally present with the child.
(3) Apologizing: By apologizing to the child for their own misbehavior, parents learn to take responsibility for their relationship with the child and transform it. The parents discuss how their behavior must have affected the child and what they would like to change in the future.
(4) Setting appropriate boundaries for children: Parents learn to set clear but flexible boundaries without judging the child.

Imaginative two-chair work is used to help parents understand emotional traps in dealing with their child and to facilitate emotionally focused processing of the child’s behavior (Dolhanty et al., 2022). This, in turn, is intended to strengthen parents’ self-efficacy and mentalization skills (understanding behavior in terms of mental states).

#### 2.5.3 Treatment fidelity

To ensure fidelity to the material as well as adequate engagement with the content of the intervention, the students, who carry out the intervention, receive training from a staff member of our institute. Furthermore, we created a manual explaining every step of the intervention in detail. During training, these guidelines and materials are discussed in a small group of approximately 10 students at a time. All activities used in the intervention are then demonstrated within the group of students. This is done to ensure that the students are equipped to carry out the intervention program. Each school visit is led by an experienced student. The students also meet shortly before the visit to discuss the implementation in advance.

### 2.6 Outcome measurements

The demographic variables recorded for parents, students and teachers are age, gender, migrant background and German language skills. Teachers are also asked how many years they have been teaching. College students are asked about their subject, semester of study and any previous medical experience, and medical field of interest. Parents are also asked about their socioeconomic status (income, highest level of education). Children are asked about their age, gender and grade. Sociodemographic data is collected through self-report in the first survey. The first survey for the teachers is at t_3_.

To assess mental health literacy children are presented with one of three case vignettes, with a different case vignette presented at each time point of the survey. The order in which each vignette is presented to each child is counterbalanced by intraindividual block randomization to avoid possible sequence effects. The three case vignettes used are self-designed cases based on the vignettes used to measure mental health literacy by Jorm et al. (1997). The case vignettes are similar to Jorm’s vignettes in that the case is a description of a situation in which mental distress or illness can be identified. However, in contrast to Jorm’s case vignettes, the case vignettes used here portray three children (Noah, Kim, Conny) rather than adults as affected. The three names were chosen to be gender neutral. In Noah’s case, symptoms of depression are described; in Kim’s case, symptoms of attention deficit hyperactivity disorder are described; and in Conny’s case, symptoms of conduct disorder are described. For each case vignette, there are two open questions and 14 closed questions. The open questions are, “What do you think is Noah’s [Kim’s/Conny’s] problem?” and “What do you think would be the best way to help Noah [Kim/Conny]?” The closed questions relate to the assessment of the severity and origin of the stress, as well as the assessment of self-help strategies in the situation described: E.g., “Do you think Noah [Kim/Conny] might have a mental health problem?” or “What do you think could improve Noah’s [Kim’s/Conny’s] situation - If Noah [Kim/Conny] stayed at home? Children respond using a simplified 3-point Likert scale (1 = Yes, 2 = Maybe, 3 = No), adapted to their cognitive and developmental level. The case vignettes were first tested for comprehension in a fourth grade-class, in which n = 22 children commented on the comprehensibility and were subsequently revised. Question wording and selection were based on theoretical considerations of the knowledge component of mental health literacy, items from a validated adult mental health literacy questionnaire (MHLq-SVa; Campos et al., 2022), and consultation with experts in the field to ensure item content validity.

An adapted German version (Sharples et al., 2017; Kaess et al., 2019) of the General Help-Seeking Questionnaire (GHSQ; Wilson et al., 2005) is administered to assess the intentional help-seeking behaviors of children and parents concerning mental health issues. Parents respond on a 7-point Likert scale ranging from 1 (extremely unlikely) to 7 (extremely likely). Children respond using a simplified 3-point Likert scale (1 = agree, 2 = neutral, 3 = strongly disagree). An example item reads, “If you [or your child] were experiencing a mental health problem (e.g., feeling stressed, angry, depressed, anxious, or worried), how likely is it that you would seek help from the following people?” The questionnaire lists 8 potential sources of help, including both informal (e.g., friends, family members) and formal (e.g., doctor, psychologist) options, as well as, “I would not seek help from anyone” and an open-ended response to specify other people not listed. For scoring purposes, the sum of the listed eight help sources is used to calculate an overall score for intentional help-seeking behavior. Higher scores indicate greater help-seeking intentions. The instrument demonstrates good to very good internal consistency (Cronbach’s *α* = 0.85) and strong test–retest reliability over a three-week period (*r* = 0.92; Wilson et al., 2005).

To assess actual help-seeking behavior, an adapted German version (Sharples et al., 2017; Kaess et al., 2019) of the Actual Help-Seeking Questionnaire (AHSQ; Rickwood and Braithwaite, 1994) is used. Children and parents are asked to tick all formal and informal sources, from a provided list of 8 potential sources (see the GHSQ), from which they have sought help in the past 6 months because they/their child experienced a mental health problem (were stressed, angry, depressed, anxious or worried). In addition, participants have the option to add a person not listed, to indicate that they did not seek help from anyone, or to indicate that they/their child did not have a mental health problem in the past 6 months.

The German translation of the Peer Mental Health Stigmatization Scale (PMHSS; McKeague et al., 2015), with a reported internal consistency of Cronbach’s *α* = 0.81, is used to assess stigmatizing attitudes toward children with mental health problems among children, students, parents and teachers. It comprises 11 items rated on a 5-point Likert scale ranging from 1 (strongly agree) to 5 (strongly disagree), for example, “I believe that children with mental health problems are dangerous.” The total stigma score is calculated by summing responses across all items, with reverse coding applied to negatively worded items, whereby a lower score indicates a higher level of stigmatization, and a higher score indicates a lower level of stigmatization.

The stigma of mental disorders among college students is assessed using the validated German version of the Opening Mind Scale for Health Care Professionals (OMS-HC; Modgill et al., 2014; Zuaboni et al., 2021). The German translation of the OMS-HC showed an acceptable internal consistency with Cronbach’s α = 0.74 (Zuaboni et al., 2021). An example item from the scale is as follows, “I am more comfortable helping a person who has a physical illness than I am helping a person who has a mental illness.” The items are then rated by the students on a 5-point Likert scale ranging from 1 (strongly agree) to 5 (strongly disagree).

To screen the mental health status of the children, the KIDSCREEN 10 Index (Ravens-Sieberer et al., 2010; Ravens-Sieberer et al., 2014), both as a self-report completed by the children and as an external proxy report provided by their parents, is used. The instrument consists of 10 items that evaluate the child’s mental health status over the past week, along with one item offering an overall assessment of their health status. It has good internal consistency, with a reported Cronbach’s α of 0.82. An example item is, “Did you feel sad?” Responses are recorded on a 5-point Likert scale ranging from 1 (not at all/never) to 5 (very much/always), with lower scores indicating poorer mental health and higher scores indicating better mental health.

We also assess parents’ mental health using the German version of the Patient Health Questionnaire-4 (PHQ-4; Kroenke et al., 2009; Löwe et al., 2010). The PHQ-4 assesses symptoms of depression and anxiety using four items rated from 0 (not at all) to 3 (almost every day), resulting in a total score of 0–12. Higher scores indicate greater severity of symptoms. The scale has good reliability (Cronbach’s α = 0.85).

Satisfaction with the prevention program is measured among the participating children, teachers and college students who deliver the program, on the basis of Anna Freud’s outcome report (Sharples et al., 2017). Children’s satisfaction with the intervention is measured with an overall evaluation of the lessons: “All in all, I found the project day” 1 (good), 2 (average), and 3 (bad). In detail, children, teachers and college students assess how much they agree with four statements. The project day: (a) “was easy to understand (for the children)”; (b) “was helpful (for the children)”; (c) “was interesting (for the children),” (d) “was fun (for the children),” rated on a 5-point Likert scale ranging from 1 (strongly agree) to 5 (strongly disagree). Children respond using a simplified 3-point Likert scale (1 = agree, 2 = neutral, 3 = strongly disagree). College students and children can indicate whether the lesson was 1 (too short), 2 (too long) or 3 (just right). The children and college students can make comments in 3 open questions about what they liked the most and least, and what else they would have liked from the project day. The teachers are asked four open-ended questions about opportunities, challenges and criticisms of the prevention project, as well as their own experiences and expertise regarding children’s mental health. Teachers are also asked if they can imagine carrying out the project on their own and by whom they would like the project to be carried out by themselves, school social workers, external staff, or others.

The Parental Reflective Functioning Questionnaire (PRFQ; Luyten et al., 2017) is used to assess mentalizing, i.e., a parent’s capacity to understand and interpret both their own and their child’s mental states. The PRFQ consists of 18 self-report items divided into three subscales: Pre-Mentalizing Modes (PM), Certainty about Mental States (CMS), and Interest and Curiosity (IC). An example item is, “I can sometimes misunderstand the reactions of my child.” The items are rated on a 7-point Likert-scale ranging from 1 (strongly disagree) to 7 (strongly agree). Subscale scores are calculated as the mean of the respective items of each subscale. Higher scores on the PM subscale indicate greater difficulties in mentalizing, whereas higher scores on the CMS and IC subscales reflect greater certainty and curiosity about the child’s mental states, respectively. The internal consistency of the scale is acceptable for the PM subscale (Cronbach’s *α* = 0.70), good for the CMS subscale (Cronbach’s *α* = 0.82), and acceptable for the IC subscale (Cronbach’s *α* = 0.75; Luyten et al., 2017).

Parental self-efficacy is assessed using the Parenting self-efficacy questionnaire (FSW; Kliem et al., 2014). Parents rate their parenting self-efficacy using 9 items on a scale ranging from 1 (strongly disagree) to 4 (strongly agree), for example, “I have the ability to set boundaries with my child.” Higher scores indicate higher levels of parental self-efficacy. Good validity and acceptable reliability were found (Cronbach’s α = 0.78–0.79).

### 2.7 Data management

The data are collected and evaluated in pseudonymized form at the Institute for Psychosocial Prevention at the Heidelberg University Hospital, Germany. This involves using a code instead of specifying names. A paper coding list links the names to this code. Access to this coding list is restricted to the study coordinator (SP) and the principal investigator (ST). Once the evaluation is complete, the coding list will be deleted by 31 December 2026 at the latest. The data will then be anonymized. This anonymized data will be stored for at least 10 years after the evaluation or the publication of a paper based on this study. Only project staff involved in data collection and analysis, such as research assistants, have access to the pseudonymized data and the final anonymized dataset. Data collection is conducted via the online survey tool SoSci Survey (Leiner, 2024). Paper-and-pencil questionnaires are also entered into the system and then securely destroyed. SoSci Survey does not claim ownership of the questionnaires and will neither use nor disclose the data for its own purposes. Confidentiality is a prerequisite for project staff in order to ensure data security. No risks are expected to arise from participation. Participation is voluntary and requires informed consent. It can be withdrawn at any time without any disadvantages. For minors, informed consent must be provided by their legal guardians.

### 2.8 Statistical analysis

Data will be analyzed using the intention-to-treat approach. Multiple imputation will be used to impute missing values. We will analyze and report on attrition. We will check for possible correlations between our variables, primary and secondary outcomes, as well as sociodemographics, and dropout at different time points. Additionally, we will report the reasons given by parents or children for withdrawal. The main analysis is a multilevel analysis due to the nested data structure (children in school classes). Both inter- and intraindividual differences and changes are analyzed. Demographic variables will be included in the data analysis model to control for group differences at baseline. In addition, we will examine a possible mediating effect of increased mental health literacy on increased help-seeking behavior through reduced stigma using multilevel mediation analysis. In addition, we will use correlational analyses as well as chi-squared tests, *t*-tests, Mann–Whitney-U-tests and *F*-tests for group comparisons and comparisons across time points. A significance level of *α* = 0.05 will be used. To validate the newly constructed mental health literacy scale, exploratory factor analysis (EFA) will be conducted, and the comparability of the three vignettes will be checked using ANOVAs. Qualitative data will be analyzed using Mayring’s content analysis using both deductive and inductive approaches (Mayring, 2015). On the one hand, the responses will be matched (deductively) to a pre-established coding scheme to indicate the extent to which the mental health problem is understood by the participating children, and the appropriateness of the help offered by the child. The children’s responses will be grouped blindly by two independent assessors. In the case of discrepancies, the scores will be discussed. On the other hand, categories will be formed (inductively) from the answers to gain more precise insight into the content of the children’s answers.

## 3 Discussion

Mental disorders are prevalent in elementary school-aged children (Morrison Gutman et al., 2015). Increased stress among children due to the COVID-19 pandemic (Ravens-Sieberer et al., 2022), long waiting times for therapy for existing mental disorders (Singer et al., 2022), numerous negative consequences of mental disorders (e.g., Shaw et al., 2012; Asselmann et al., 2018), and low mental health literacy among children and parents (Reardon et al., 2017; Tharaldsen et al., 2017) highlight the urgent need for early prevention approaches to improve mental health in schools. However, to date, most prevention approaches have focused only on older children or parents (Ma et al., 2023a). Therefore, the aim of this study is the evaluation of the prevention program “Talking mental health” tailored for 8–10-year-old school children in Germany. This program teaches children in a playful way what mental health is and how to distinguish between big and small feelings. It helps children understand when they need to seek help (when big feelings are present), from whom they should seek help, and when their coping strategies (toolbox), e.g., reading, journaling, and bathing, are sufficient (when small feelings are present). Children learn to talk about how they feel and learn to listen. Parents learn where to seek help, what services are available for specific problems and how to talk to their children about their feelings. These activities aim to increase mental health literacy, reduce stigma and encourage help-seeking behavior. The project also goes a step further by offering parents psychological counseling and evidence-based parenting training.

The strength of this prevention program is that it can be carried out by student volunteers. This means that teachers and school social workers do not incur any additional costs or burdens. Moreover, both groups can participate in the intervention and build on it. If effective, this would provide a scientifically tested prevention program for elementary schools that could be implemented cost-effectively on a larger scale. As a result, families may be more likely to take advantage of the care structures in place, thus reducing the risk of chronicity.

In addition to a universal prevention approach, we also offer an indicative prevention approach for particularly burdened families. In this way, the project also contributes to reducing psychological stress in children and their families.

It is often difficult to carry out randomization in the school context, which is why many evaluation studies in this area only use a quasi-experimental design. However, our design, with a waiting list control group, seems to be an appropriate and ethically acceptable approach. Nevertheless, there are a number of methodological and practical challenges associated with this study and with school-based prevention for children in general.

We developed our own vignettes, as we were not aware of any established instruments for measuring mental health literacy in children aged 8–10 years. The stigma measure (PMHSS; McKeague et al., 2015) was tested only with children aged 9 + years. In agreement with the authors, we also used it with 8-year-olds. We also support the children by having at least one project worker present during the surveys. The children can ask questions, and any unfamiliar words are explained.

In addition, the planned intervals between surveys vary slightly between schools because of holidays or internal school processes. However, we ensure that there is at least 1 week between survey 1 and 2 and at least 6 weeks between survey 1 and 3.

This first evaluation of the prevention program measures effects only after 6 weeks and therefore captures only short-term outcomes. If there is early evidence of effectiveness, then long-term effects should also be part of the evaluation in future studies.

Past studies have shown that children may not benefit equally from interventions designed to promote well-being. In one study, some children did not benefit from a mindfulness-based intervention, and others even showed a decrease in well-being (Kuyken et al., 2022). The authors argued that mindfulness exercises may have directed attention to unpleasant emotional states, that teachers may not have implemented the intervention adequately, or that additional strategies for coping with stress might have been necessary (Kuyken et al., 2022). In our “Talking mental health” school lessons, discussions about feelings and mental health may also potentially reactivate existing stress or unpleasant emotional states in children. Therefore, during lessons, we continuously monitor the children’s stress levels using the “mood barometer.” Children always have the option to leave the room together with the teacher or a trained student conducting the lesson. The students delivering the lessons are specifically trained to handle such situations. Additionally, a toolbox for dealing with different emotions is developed collaboratively with the children. At the end of the program, parents are offered the opportunity to attend a psychological consultation. During this meeting, any open questions can be addressed, and parents and children can be referred for further support if needed. Overall, the benefits of participating in the lessons clearly outweigh any potential risks.

In our study, we primarily relied on self-reports. In this young age group, such reports may be influenced by social desirability or other response biases (Soneson et al., 2025; Wang and Zang, 2025). Mental health stigma (Kaushik et al., 2016) or concerns regarding data security (Soneson et al., 2025) may prevent children from openly reporting whether they are seeking help or have mental health difficulties. Similar issues, including stigmatization and shame, have also been described in the context of self-reported bullying assessments (Furlong et al., 2010). In accordance with the recommendations by Wang and Zang (2025), we used neutral wording and avoided leading questions. Prior to the interview, we informed the children that their answers would be treated as strictly confidential and recorded using a number–letter code instead of their names. We also reminded them that there were no right or wrong answers. We did not include social desirability detection items or validity screening items, which should be considered in future research (Cornell et al., 2012; Wang and Zang, 2025).

In addition, only schools in the Heidelberg area are included in this first evaluation study (convenience sampling method). The socioeconomic status in Heidelberg is above German average. Therefore, we consider the influence of socioeconomic status and migration background in our evaluation. It should be noted that, so far, 12 of the 28 schools contacted have expressed interest in participating in the study. Possible differences between participating and non-participating schools will be examined and reported after data collection in order to identify possible biases. When interpreting the findings, it is important to note that the results may not be representative or generalizable. This study should therefore be understood as an initial evaluation in the German-speaking context. Future research should implement the intervention in more rural areas and different countries.

Due to the small number of recruited schools, and for reasons of model parsimony and statistical power, we have decided not to include the school as an additional level in our main model. In exploratory analyses, we will examine variance at the school level to determine whether these differences can explain treatment effects. Due to data governance regulations, specific information at the school level, such as exact distributions of migration background and socioeconomic status, is currently not available. However, publicly accessible data from the relevant local administrative districts may serve as a reference.

This study will provide valuable insights into the effectiveness of the “Talking mental health” program in increasing mental health literacy and help-seeking behavior and in decreasing stigma. In addition, analyzing satisfaction ratings and reasons for withdrawal will offer important information regarding the acceptance and future implementation of such programs.

### 3.1 Trial status

The data were collected from children in 30 classes. Data collection and project implementation are planned for an additional 6 classes until the end of July 2025. Recruitment of parents for the second part of the study and intervention (parent training) is ongoing. One parent group has already taken place.

## 4 Ethics and dissemination

This research was approved by the Ethics Committee of the Faculty of Behavioral and Cultural Studies, Heidelberg University (AZ Tau 2023 5/1) in November 2023 (approval of amendment July 2024). Prior to participation in the study, teachers, parents, children and students are informed verbally and in writing about the procedure and content of the study. They are informed about data storage, data transfer, data publication and data protection. All the participants provided or will provide written informed consent. Parents provide or will provide informed consent for their children.

The following measures are included in the dissemination plan for the Talking mental health program: (a) report the findings regarding the efficacy of the intervention in peer-reviewed journals, (b) revise the structure of the program based on the feedback collected, (c) establish further theoretical and practical training for college students at other universities and possibly also address teachers and social workers to enable a wider spread implementation, (d) potentially translate the material (manual, worksheets, presentation and video) into more languages to provide access in other countries.

## Footnotes

1
https://www.randomizer.org/

2
https://www.youtube.com/watch?v=nCrjevx3-Js

3
https://www.youtube.com/watch?v=jNhmBn-qICo
